# Association of Attendance Format with Learning Gain and National Examination Performance in a HyFlex Preparatory Course for Pharmacy Students

**DOI:** 10.3390/pharmacy14060135

**Published:** 2026-09-17

**Authors:** Yukinori Nagakura, Fumiko Yamaki, Hiroshi Saimaru, Kou Hiroya, Koji Ichinose

**Affiliations:** 1 Research Center for Pharmaceutical Career Education, Faculty of Pharmacy, Musashino University, 1-1-20 Shinmachi Nishitokyo-shi, Tokyo 202-8585, Japan; f-yamaki@musashino-u.ac.jp (F.Y.); hirosai@musashino-u.ac.jp (H.S.); 2 Faculty of Pharmacy, Musashino University, 1-1-20 Shinmachi Nishitokyo-shi, Tokyo 202-8585, Japan; k_hiroya@musashino-u.ac.jp (K.H.); ichinose@musashino-u.ac.jp (K.I.)

**Keywords:** national examination for pharmacists, HyFlex course, attendance format, learning outcomes, pharmacy education

## Abstract

Attendance format may be associated with the learning-support effects of preparatory courses for the National Examination for Pharmacists (NEP). This retrospective observational study examined associations of self-selected attendance format with course-period learning gain and subsequent NEP performance in a four-month HyFlex course for sixth-year pharmacy students. Students received the same course content and examinations and were classified into face-to-face (F2F), live online (LO), or mixed (MIX) attendance groups. Course-period learning gain was defined as the difference in examination score percentage between the post-course and pre-course examinations. The F2F group showed greater learning gain than the LO and MIX groups. Although the NEP pass rate was higher in the F2F group, the difference among groups did not reach statistical significance in the primary analysis (*p* = 0.086). Similar findings were observed in a sensitivity analysis. In conclusion, F2F attendance was associated with greater course-period learning gain than LO or MIX attendance. However, this advantage was not clearly reflected in NEP outcomes, indicating that course-period learning gain alone may not fully explain final examination performance and continued post-course support may be needed. Because the attendance format was self-selected, these findings should not be interpreted as evidence of a causal effect.

## 1. Introduction

In Japan, the National Examination for Pharmacists (NEP) is administered annually over two days in late February to assess learning outcomes accumulated over six-year pharmacy education programs and to determine whether students have acquired the knowledge and skills required of pharmacists. The examination covers physics, chemistry, biology, hygiene, pharmacology, pharmaceutics, pathophysiology/pharmacotherapy, laws/regulations/ethics, and practical pharmacy and requires not only foundational knowledge but also the ability to integrate and apply pharmaceutical knowledge in clinical and social contexts. In recent years, the overall pass rate has remained at approximately 70% [1]. Internationally, pharmacist licensure examination performance has been recognized as an important issue in pharmacy education. In the United States, declining first-time pass rates on the North American Pharmacist Licensure Examination have prompted discussion about student preparedness, institutional factors, curricular design, and the effectiveness of licensure examination preparation programs [2,3,4,5]. In line with these international concerns, Japanese pharmacy education also requires effective strategies to help students reach the academic level necessary to pass the NEP, particularly because NEP performance varies across pharmaceutical colleges and may reflect both students’ academic standing at admission and the educational support provided during the curriculum [1,6,7,8].

The NEP requires students not only to recall foundational knowledge but also to integrate concepts across multiple subject areas and apply them to clinically and socially relevant problems [9]. Therefore, in the final stage of six-year pharmacy education, educational strategies are needed to help students organize and integrate the broad range of pharmaceutical knowledge they have acquired and to support their attainment of the academic level necessary to pass the examination. NEP preparatory courses represent a typical form of such learning support, and improving their educational effectiveness is therefore important.

One factor that may influence learning outcomes is the mode of attendance or instructional delivery. Previous reviews and meta-analyses have compared the educational effectiveness of face-to-face, online, and blended instruction; however, their conclusions have not been fully consistent [10,11,12,13]. These studies suggest that the effectiveness of online or blended instruction may depend on multiple conditions, including learner characteristics, course content, learning duration, instructional design, and outcome measures. In long-term, high-intensity educational settings such as NEP preparatory courses, students must maintain continuous learning behaviors and integrate knowledge across multiple subject areas. Therefore, the influence of attendance mode on both course-period learning gain and subsequent NEP performance remains insufficiently examined.

At the Faculty of Pharmacy, Musashino University, a four-month NEP preparatory course is offered to sixth-year students immediately before the NEP. The course is delivered in a HyFlex format, in which instructors teach students in the classroom while simultaneously streaming the same lectures online, allowing students to choose either face-to-face (F2F) or live online (LO) attendance for each class session [14]. Pre- and post-course examinations using the same format as the NEP are administered in the classroom to objectively evaluate students’ learning outcomes. Our previous study, which included students enrolled in FY2023 and FY2024, showed that attendance format was associated with examination-based learning gain and that the F2F group had greater learning gain than the other groups [15]. However, that study was limited to course-period outcomes and could not evaluate whether attendance format was associated with subsequent NEP performance because the two-year sample was insufficient for an analysis of the final licensure-examination outcome. Thus, whether the observed course-period advantage was reflected in NEP performance remained unknown.

Using data from three academic years, FY2023 to FY2025, the present study extends our previous two-year analysis by increasing the sample size and examining both course-period learning gain and subsequent NEP performance. Specifically, we compared overall learning gain, defined as the difference in examination score percentage between the post-course and pre-course examinations, and NEP pass or fail/non-attendance outcomes among the F2F, live online (LO), and mixed (MIX) attendance groups. The primary analysis included students who completed both examinations and met the predefined 90% overall course-attendance criterion. To assess the potential influence of this criterion, we also conducted a sensitivity analysis that included students who completed both examinations but did not meet the 90% attendance criterion. By jointly evaluating an intermediate course-period outcome and the final licensure-examination outcome, this study addresses the knowledge gap remaining after our previous report.

## 2. Materials and Methods

### 2.1. Study Design and Setting

This retrospective observational study was conducted using data from HyFlex NEP preparatory courses offered to sixth-year pharmacy students from FY2023 to FY2025. Students voluntarily selected either F2F or LO attendance for each class session and were subsequently classified into F2F, LO, or MIX attendance groups according to their primary attendance format. All students received the same NEP-related lecture content through the same HyFlex course format. The study examined the association between attendance format and learning outcomes, including learning gains during the course period and performance on the NEP.

### 2.2. NEP Preparatory Course Structure and Delivery

At the Faculty of Pharmacy, Musashino University, a four-month preparatory course for the NEP was conducted for sixth-year students in each academic year from FY2023 to FY2025, from September to January. The course covered physics, chemistry, biology, hygiene, pharmacology, pharmaceutics, pathophysiology and pharmacotherapy, laws/regulations/ethics, and practical pharmacy and was designed to support the acquisition of knowledge and theoretical understanding required of pharmacists. Each class session was generally 90 min long. The course schedule and the number of lectures by subject are shown in Table 1. Lectures were delivered in a HyFlex format by instructors from Igaku Academy (Saitama, Japan). Instructors delivered lectures to F2F participants in the classroom while simultaneously streaming the lectures to LO participants via Zoom (Zoom Video Communications, Inc., San Jose, CA, USA).

### 2.3. Pre- and Post-Course Examination Procedures

The pre-course examination was administered in September before the start of the preparatory course, and the post-course examination was administered in January after course completion. Examination questions were prepared by Igaku Academy (Saitama, Japan), a preparatory school for the NEP. Both examinations followed the same format, subject areas, question structure, and examination time as the NEP and were conducted over two consecutive days. All students took the examinations in the same classroom according to the same schedule. Each examination consisted of 345 multiple-choice questions and was administered as a written mark-sheet examination. The subject allocation was as follows: physics, 20 questions; chemistry, 20 questions; biology, 20 questions; hygiene, 40 questions; pharmacology, 40 questions; pharmaceutics, 40 questions; pathophysiology and pharmacotherapy, 40 questions; laws, regulations, and ethics, 30 questions; and practical pharmacy, 95 questions. Each question was scored as one point, and overall and subject-specific scores were converted into score percentages.

### 2.4. Participants and Group Classification

The study population comprised sixth-year students at the Faculty of Pharmacy, Musashino University, who participated in the NEP preparatory course in FY2023 (*n* = 128), FY2024 (*n* = 121), or FY2025 (*n* = 125), for a total of 374 students. Attendance records for all class sessions between the pre- and post-course examinations were obtained from signed attendance sheets for F2F participants and Zoom viewing logs for LO participants. The primary analysis included students who completed both examinations and had an overall course-attendance rate of at least 90%. The sensitivity analysis additionally included students who completed both examinations but had an overall course-attendance rate below 90%. Students who did not complete either the pre- or post-course examination were excluded from both analyses because learning gain could not be calculated. Eligible students were classified according to their primary attendance format. Students who attended at least 75% of their sessions in the F2F format were assigned to the F2F attendance group, whereas those who attended at least 75% of their sessions in the live online format were assigned to the LO attendance group. Students who did not reach 75% attendance in either format were assigned to the MIX attendance group. The 75% threshold was used to identify students with relatively clear exposure to a given attendance format.

### 2.5. Evaluation of Learning Outcomes

Course-period learning outcomes were evaluated using learning gain, calculated as the post-course examination score percentage minus the pre-course examination score percentage. Overall learning gain, based on overall score percentage, was defined as the primary outcome and was used for statistical significance testing. Subject-specific learning gains, based on subject-specific score percentages, were reported as reference values and were not subjected to statistical significance testing because repeated testing across multiple subjects could increase the risk of false-positive findings.

### 2.6. Evaluation of Performance on the NEP

Because individual NEP scores were not publicly disclosed, NEP performance was evaluated using pass or fail/non-attendance status. For each student included in the analysis, performance on the NEP administered approximately one month after course completion was classified as pass or fail/non-attendance. Students who did not take the NEP because they had failed to graduate were classified as fail/non-attendance; all non-attendance cases were attributable to non-graduation. The pass rate was then calculated for each attendance-format group.

### 2.7. Statistical Analysis

Statistical analyses were performed separately for the primary and sensitivity analysis populations. Comparisons were conducted among the F2F, LO, and MIX attendance groups. Overall learning gain, calculated as the post-course examination score percentage minus the pre-course examination score percentage, was compared among the three groups using one-way analysis of variance (ANOVA), followed by the Tukey–Kramer post hoc test for multiple comparisons. For each pairwise comparison, the estimated mean difference in overall learning gain and its 95% confidence interval (CI) were reported. NEP performance, classified as pass or fail/non-attendance, was compared using Fisher’s exact test, with Bonferroni correction applied for post hoc pairwise comparisons. Baseline group differences were assessed using one-way ANOVA for pre-course examination score percentages and Fisher’s exact test for sex distribution. Pre- and post-course examination score percentages were compared using paired Student’s *t*-tests. Cohen’s d was calculated as an effect size for within-group and between-group comparisons, as appropriate. Statistical analyses were performed using EZR (Jichi Medical University, Saitama, Japan) and BellCurve for Excel (Social Survey Research Information Co., Ltd., Tokyo, Japan). A two-sided *p* value of less than 0.05 was considered statistically significant.

## 3. Results

### 3.1. Participant Classification by Attendance Format

Of the 374 students in the study population, 355 completed both the pre- and post-course examinations, whereas 19 did not complete one or both examinations and were excluded from the analysis. Among students included in the analysis who completed both examinations, 301 had an overall attendance rate of at least 90%, whereas 54 had an overall attendance rate below 90%. In the primary analysis, 301 students who met the 90% overall attendance criterion were classified according to the attendance-format criteria into three groups: the F2F attendance group (*n* = 38), MIX attendance group (*n* = 64), and LO attendance group (*n* = 199). In the sensitivity analysis, 355 students, including those with an overall attendance rate below 90%, were classified according to the same attendance-format criteria into three groups: the F2F attendance group (*n* = 43), MIX attendance group (*n* = 69), and LO attendance group (*n* = 243).

### 3.2. Baseline Characteristics Before the Preparatory Course

Baseline characteristics before the start of the preparatory course are shown in Table 2. In the primary analysis population, no statistically significant differences were observed among the three groups in overall pre-course examination score percentages (one-way ANOVA, *p* = 0.964) or sex distribution (Fisher’s exact test, *p* = 0.326). Similarly, in the sensitivity analysis population, no statistically significant differences were observed among the three groups in overall pre-course examination score percentages (one-way ANOVA, *p* = 0.925) or sex distribution (Fisher’s exact test, *p* = 0.417).

### 3.3. Overall Pre- and Post-Course Examination Score Percentages

In the primary analysis population (*n* = 301), the overall post-course examination score percentage was significantly higher than the pre-course examination score percentage by paired Student’s *t*-test (*p* < 0.001; Cohen’s d = 1.62). In the sensitivity analysis population (*n* = 355), which included students who completed both examinations but did not meet the 90% attendance criterion, the overall post-course examination score percentage was also significantly higher than the pre-course examination score percentage by paired Student’s *t*-test (*p* < 0.001; Cohen’s d = 1.54). Pre- and post-course examination score percentages are summarized in Table 3. These findings indicate that overall examination performance improved during the course period in both analysis populations.

### 3.4. Comparison of Learning Gain Among Attendance-Format Groups

In the primary analysis, overall learning gain was greater in the F2F attendance group than in the MIX and LO attendance groups. One-way ANOVA showed a significant difference among the three groups: F(2, 298) = 3.83, *p* = 0.023, η^2^ = 0.025. Tukey–Kramer post hoc testing showed significant differences between F2F and LO (mean difference, 3.1 percentage points; 95% CI, 0.93 to 5.27 percentage points; *p* = 0.022; d = 0.46) and between F2F and MIX (mean difference, 3.3 percentage points; 95% CI, 0.92 to 5.68 percentage points; *p* = 0.040; d = 0.58), but not between MIX and LO (mean difference, −0.2 percentage points; 95% CI, −1.50 to 1.89 percentage points; *p* = 0.976; d = 0.03). In the sensitivity analysis, overall learning gain also differed significantly among the three groups: F(2, 352) = 4.74, *p* = 0.009, η^2^ = 0.026. Tukey–Kramer post hoc testing showed a significant difference only between F2F and LO (mean difference, 3.5 percentage points; 95% CI, 1.55 to 5.50 percentage points; *p* = 0.006; d = 0.50); differences between F2F and MIX (mean difference, 3.1 percentage points; 95% CI, 0.77 to 5.33 percentage points; *p* = 0.062; d = 0.51) and between MIX and LO (mean difference, 0.4 percentage points; 95% CI, −1.26 to 2.21 percentage points; *p* = 0.870; d = 0.07) were not statistically significant. The effect estimates should be interpreted cautiously because the groups were unequal in size, particularly the relatively small F2F group. Overall and subject-specific learning gains by attendance format are shown in Table 4.

### 3.5. Performance on the NEP

The numbers of students who passed and those classified as fail/non-attendance, as well as the pass rate in each group, are shown in Table 5. In the primary analysis, differences in pass rates among the three groups did not reach statistical significance (Fisher’s exact test, *p* = 0.086). In the sensitivity analysis, although Fisher’s exact test showed a significant difference among the three groups (*p* = 0.043), Bonferroni’s post hoc tests showed no statistically significant differences in any pairwise comparison (F2F vs. LO, *p* = 0.074; F2F vs. MIX, *p* = 0.078; MIX vs. LO, *p* = 1.000). These findings suggest that the association between attendance format and NEP performance was less consistent than that observed for course-period learning gain.

## 4. Discussion

This study examined whether attendance format was associated with course-period learning gain and subsequent NEP performance in a four-month HyFlex preparatory course for pharmacy students. The findings partly support our working hypothesis: the F2F attendance group showed greater improvement from the pre-course to post-course examinations than the LO and MIX attendance groups, whereas this advantage was not clearly reflected in NEP performance. These results suggest that attendance format may influence learning gain during the structured course period, but that final examination outcomes are likely shaped by additional factors beyond course-period improvement.

In this study, the F2F attendance group showed greater learning gain, defined as the difference in examination score percentage between the post-course and pre-course examinations, than the LO and MIX attendance groups. This finding is consistent with our previous study based on data from FY2023 and FY2024 [15]. The preparatory course was conducted over four months in a continuous, high-intensity learning environment for sixth-year students preparing for the NEP. In this context, learning gain may have depended not only on students’ understanding of the course content but also on their ability to maintain a regular learning rhythm, sustain concentration, and engage in continuous learning behaviors [16,17,18]. F2F attendance may have supported these behaviors by providing a fixed time and place for participation, direct interaction with instructors and classmates, and opportunities to observe peers’ learning behaviors [19,20,21]. The classroom setting may also have reduced distractions and facilitated immediate clarification of difficult points during or after lectures. These structured features may partly explain why the advantage of F2F attendance was observed mainly in course-period learning gain.

Because the primary analysis included only students who met the predefined 90% attendance criterion, the attendance-based inclusion criterion may have influenced the results. Students who did not meet this criterion may have differed from those included in the primary analysis in motivation, learning persistence, self-regulated learning ability, or learning environment. To examine the potential impact of this criterion, we performed a sensitivity analysis that additionally included students who completed both examinations but did not meet the 90% attendance criterion. The results were generally consistent with those of the primary analysis, suggesting that the main finding regarding learning gain across attendance formats was robust to the inclusion of students with lower attendance. Nevertheless, low attendance remains an important issue for improving the learning-support effects of the preparatory course [4]. Further studies are therefore needed to identify factors associated with lower attendance and to develop support strategies for students at risk of insufficient participation.

This study examined the association between attendance format and NEP performance as a final learning outcome, which had not been analyzed in our previous study [15]. Using data from three academic years, we found that the greater course-period learning gain observed in the F2F attendance group was not clearly reflected in NEP outcomes. Although the F2F attendance group showed greater learning gain than the other groups, the three-group comparison of NEP pass rates did not reach statistical significance. This discrepancy may be explained by several factors. First, the pre- and post-course examinations used to calculate learning gain were mock examinations modeled on the NEP, rather than the actual NEP. Second, because approximately one month elapsed between course completion and the NEP, learning during this interval may have influenced final performance. Third, students’ physical and psychological condition on the day of the actual examination may have affected performance under pressure that was not fully reproduced in the mock examinations. These factors may explain why the greater learning gain observed in the F2F attendance group did not translate into a statistically significant advantage in NEP performance.

These findings indicate that course-period learning gain and final licensure examination performance should be interpreted as related but distinct outcomes. Learning gain based on pre- and post-course examinations reflects improvement during the structured course period, whereas NEP performance is also influenced by post-course self-study, test-taking readiness, and physical and psychological condition at the time of the actual examination. Because course-period learning gains do not automatically guarantee NEP success, sustaining both the quality and volume of study efforts during the post-course interval, together with maintaining their physical and mental readiness, appears to be essential for final success. Future preparatory courses may need to combine course-period instruction with structured post-course support, including individualized learning plans, progress monitoring, review of weak subject areas, and guidance on examination readiness.

Based on the present findings, several points should be considered when designing HyFlex NEP preparatory courses. The greater learning gain observed in the F2F group represents an association and should not be interpreted as proof that F2F attendance itself caused the improvement. Attendance format was self-selected, and the students who chose F2F attendance may have differed systematically from those choosing LO or MIX attendance. For example, they may have had stronger motivation, a greater preference for external structure or social interaction, different self-regulated learning skills, a lower commuting burden, or a more favorable living and learning environment. Previous HyFlex research has shown that attendance choices may be related to self-regulatory and motivational factors, commuting distance, work–life constraints, and students’ perceptions of how they learn best [22]. These characteristics could have influenced both attendance-format choice and academic performance, thereby inflating the apparent F2F advantage. Although the groups did not differ significantly in pre-course examination scores or sex distribution, these baseline comparisons do not eliminate residual confounding by unmeasured learner characteristics. HyFlex courses also offer potential advantages, including flexibility in time and place, reduced commuting burden, learner autonomy, and improved access to learning opportunities [23,24,25]. Therefore, attendance-format selection and support should consider individual learner characteristics and learning conditions, such as self-regulated learning ability, social presence, technological conditions, commuting time, and access to appropriate instructional and learning support [26,27]. Future prospective studies should measure these characteristics directly and use study designs or analytical approaches that better address selection effects.

A strength of this study is that learning outcomes among attendance-format groups were compared under relatively controlled conditions in a HyFlex NEP preparatory course. All groups received the same NEP-related lecture content through the same HyFlex delivery format and followed a unified schedule within each academic year, thereby reducing potential confounding related to differences in course content, instructors, or learning schedule [28]. Learning outcomes were assessed using objective measures: course-period learning gain, calculated from pre- and post-course examination score percentages, and NEP performance, classified as pass or fail/non-attendance status. This approach may have reduced biases associated with subjective measures, such as self-reported questionnaires or self-assessments [29,30]. In addition, all students took the pre- and post-course examinations in the same classroom and according to the same schedule, which may have minimized differences in testing conditions and reduced opportunities for academic dishonesty that can be a concern in online assessments [31].

This study has several limitations. First, the retrospective observational design and self-selected attendance format preclude causal inference. Unmeasured learner characteristics may have influenced both attendance choice and learning outcomes; thus, the observed F2F advantage may partly reflect residual confounding. Second, group sizes were substantially unequal, with relatively few students in the F2F group compared with the LO group, reducing the precision and generalizability of F2F-related estimates. Third, because individual NEP scores were unavailable, NEP performance was assessed only as pass or fail/non-attendance, which may have reduced outcome sensitivity. Fourth, this single-institution study may not generalize to settings with different student populations, course implementation, resources, support structures, or HyFlex environments [32]. Future prospective multicenter studies should collect relevant learner-characteristic data and use designs or analytical approaches that better address selection effects.

## 5. Conclusions

This study examined associations of self-selected attendance format with course-period learning gain and NEP performance in a HyFlex preparatory course. F2F attendance was associated with greater learning gain than LO or MIX attendance, and this pattern was generally consistent in the sensitivity analysis that included students who did not meet the 90% attendance criterion. However, the course-period advantage was not clearly reflected in NEP performance. These findings suggest that F2F attendance may be beneficial for supporting learning during the structured course period, while HyFlex support should remain responsive to individual learning needs and conditions. As the attendance format was self-selected, further prospective multicenter studies are warranted to confirm the observed association and clarify the independent contribution of attendance format to learning outcomes.

## Figures and Tables

**Table 1:** Subject composition of the NEP preparatory course and course/examination schedule.

Academic Year	2023	2024	2025
**Course Period**	19 September–16 January	23 September–10 January	22 September–9 January
**Number of lectures by subject**			
Physics	32	28	24
Chemistry	32	32	28
Biology	28	28	28
Hygiene	28	24	28
Pharmacology	36	32	28
Pharmaceutics	32	24	28
Pathophysiology/Pharmacotherapy	32	32	28
Laws/Regulations/Ethics	20	16	16
Practical Pharmacy	24	20	24
Total	264	236	232
**Examination schedule**			
Pre-course examination	14–15 September	19–20 September	18–19 September
Post-course examination	18–19 January	16–17 January	15–16 January
National examination schedule	17–18 February	22–23 February	21–22 February

**Table 2:** Baseline characteristics of the analysis population before the preparatory course.

	Primary Analysis Population			Sensitivity Analysis Population Primary Analysis Population Plus Students with <90% Attendance		
	F2F	MIX	LO	F2F	MIX	LO
Total: *n*	38	64	199	43	69	243
Male: *n*	13	13	54	14	16	75
(%)	(34.2%)	(20.3%)	(27.1%)	(32.6%)	(23.2%)	(30.9%)
Female: *n*	25	51	145	29	53	168
(%)	(65.8%)	(79.7%)	(72.9%)	(67.4%)	(76.8%)	(69.1%)
Subject (Maximum score)	Pre-course examination score percentage (%)					
Overall (345)	45.6 ± 9.8	45.1 ± 10.0	45.1 ± 10.6	45.1 ± 9.6	44.7 ± 10.1	45.2 ± 10.7
Physics (20)	36.3 ± 15.3	37.7 ± 12.8	37.5 ± 13.4	36.4 ± 15.0	37.5 ± 12.4	37.9 ± 13.4
Chemistry (20)	39.1 ± 13.2	37.5 ± 12.9	37.7 ± 13.3	39.7 ± 13.7	37.6 ± 13.1	37.4 ± 13.6
Biology (20)	33.3 ± 14.1	35.9 ± 14.9	34.2 ± 14.1	32.7 ± 13.6	35.2 ± 15.0	34.3 ± 14.0
Hygiene (40)	43.0 ± 10.0	45.7 ± 13.7	44.9 ± 13.7	42.5 ± 9.6	45.3 ± 13.5	45.3 ± 14.1
Pharmacology (40)	48.4 ± 13.9	46.1 ± 16.6	46.2 ± 16.2	47.3 ± 14.1	45.5 ± 16.8	46.1 ± 16.2
Pharmaceutics (40)	45.1 ± 13.3	44.1 ± 11.3	42.2 ± 12.7	43.7 ± 13.6	44.0 ± 11.2	42.8 ± 12.8
Pathophysiology /Pharmacotherapy (40)	42.3 ± 12.7	39.4 ± 12.5	40.6 ± 13.8	41.3 ± 12.3	39.2 ± 12.1	40.9 ± 13.8
Laws/Regulations /Ethics (30)	55.6 ± 12.2	54.0 ± 11.5	54.9 ± 11.4	56.0 ± 11.6	53.5 ± 11.8	54.7 ± 11.7
Practical Pharmacy (95)	49.9 ± 12.9	49.7 ± 10.7	50.3 ± 12.2	49.7 ± 12.9	48.8 ± 11.1	50.2 ± 12.4

Pre-course examination score percentages are presented as mean ± standard deviation. The sensitivity analysis population consisted of the primary analysis population plus students who completed both the pre- and post-course examinations but did not meet the 90% overall attendance criterion. F2F: face-to-face attendance; MIX: mixed attendance; LO: live online attendance.

**Table 3:** Pre- and post-course examination score percentages.

	Pre	Post	*p* Value	Cohen’s d (95% CI)
Primary analysis population (*n* = 301)	45.2 ± 10.3	62.7 ± 11.3	<0.001	1.62 (1.55–1.69)
Sensitivity analysis population (*n* = 355)	45.1 ± 10.5	62.2 ± 11.7	<0.001	1.54 (1.47–1.60)

Pre- and post-course examination score percentages are presented as mean ± standard deviation. *p* values were calculated using paired Student’s *t*-tests. The sensitivity analysis population consisted of the primary analysis population plus students who completed both the pre- and post-course examinations but did not meet the 90% overall attendance criterion. Pre: pre-course examination; Post: post-course examination.

**Table 4:** Overall and subject-specific learning gains by attendance format.

Subject (Maximum Score)	Primary Analysis Population			Sensitivity Analysis Population Primary Analysis Population Plus Students with <90% Attendance		
	F2F (%)	MIX (%)	LO (%)	F2F (%)	MIX (%)	LO (%)
Overall (345)	20.3 ± 5.9	17.0 ± 5.6	17.2 ± 7.0	20.1 ± 5.7	17.0 ± 6.2	16.6 ± 7.3
Physics (20)	14.9 ± 17.7	13.0 ± 16.8	12.4 ± 15.4	14.1 ± 17.3	12.6 ± 16.6	12.1 ± 15.3
Chemistry (20)	14.7 ± 14.7	7.3 ± 15.5	7.2 ± 14.8	12.4 ± 16.3	7.2 ± 15.9	7.2 ± 14.9
Biology (20)	23.0 ± 15.3	15.4 ± 16.4	16.1 ± 16.2	21.7 ± 14.9	15.4 ± 16.0	16.1 ± 16.1
Hygiene (40)	25.8 ± 11.7	19.1 ± 9.8	20.8 ± 12.7	25.9 ± 11.0	19.1 ± 10.2	20.0 ± 12.9
Pharmacology (40)	24.3 ± 9.2	22.7 ± 10.5	23.0 ± 12.5	24.4 ± 8.9	22.3 ± 10.8	22.1 ± 12.6
Pharmaceutics (40)	16.3 ± 13.9	15.0 ± 12.1	15.4 ± 12.2	16.8 ± 13.6	14.9 ± 13.3	14.3 ± 12.8
Pathophysiology/Pharmacotherapy (40)	22.8 ± 10.9	22.6 ± 10.9	20.3 ± 10.5	22.8 ± 10.4	22.4 ± 11.1	19.4 ± 12.6
Laws/Regulations /Ethics (30)	16.8 ± 13.7	15.2 ± 12.9	17.2 ± 12.8	16.4 ± 13.5	15.6 ± 13.5	16.3 ± 13.0
Practical Pharmacy (95)	19.8 ± 9.3	16.0 ± 8.5	15.9 ± 10.1	19.7 ± 9.5	16.4 ± 8.8	15.5 ± 10.3

Values are presented as mean ± standard deviation. Learning gain was calculated as the post-course examination score percentage minus the pre-course examination score percentage. The sensitivity analysis population consisted of the primary analysis population plus students who completed both the pre- and post-course examinations but did not meet the 90% overall attendance criterion. F2F: face-to-face attendance; MIX: mixed attendance; LO: live online attendance.

**Table 5:** NEP performance and pass rate by attendance format.

	Primary Analysis Population			Sensitivity Analysis Population Primary Analysis Population Plus Students with <90% Attendance		
	F2F	MIX	LO	F2F	MIX	LO
Pass (*n*)	36	51	161	41	55	197
Fail/non-attendance (*n*)	2	13	38	2	14	46
Pass rate (%)	94.7	79.7	80.9	95.3	79.7	81.1

Values are presented as numbers or pass rates (%). The sensitivity analysis population consisted of the primary analysis population plus students who completed both the pre- and post-course examinations but did not meet the 90% overall attendance criterion. F2F: face-to-face attendance; MIX: mixed attendance; LO: live online attendance.

## Data Availability

The data presented in this study are available from the corresponding author upon reasonable request. The data are not publicly available because they contain students’ academic performance and attendance records and are subject to privacy and ethical restrictions. Access requests will be considered in accordance with institutional ethical requirements and applicable privacy regulations.
